# Advancing Dielectric and Ferroelectric Properties of Piezoelectric Polymers by Combining Graphene and Ferroelectric Ceramic Additives for Energy Storage Applications

**DOI:** 10.3390/ma11091553

**Published:** 2018-08-28

**Authors:** Saira Ishaq, Farah Kanwal, Shahid Atiq, Mahmoud Moussa, Umar Azhar, Muhammad Imran, Dusan Losic

**Affiliations:** 1 Institute of Chemistry, University of the Punjab, Lahore 54590, Pakistan; saira_chem@yahoo.com (S.I.); farahkchem@yahoo.com (F.K.); imran_inorganic@yahoo.com (M.I.); 2 School of Chemical Engineering, The University of Adelaide, Adelaide, SA 5005, Australia; saira_chem@yahoo.com (S.I.); mahmoud.moussa@adelaide.edu.au (M.M.); umar_azhar@hotmail.com (U.A.); dusan.losic@adelaide.edu.au (D.L.); 3 The ARC Research Hub for Graphene Enabled Industry Transformation, The University of Adelaide, Adelaide, SA 5005, Australia; saira_chem@yahoo.com (S.I.); mahmoud.moussa@adelaide.edu.au (M.M.); dusan.losic@adelaide.edu.au (D.L.); 4 Centre of Excellence in Solid State Physics, University of the Punjab, Lahore 54590, Pakistan; satiq.cssp@pu.edu.pk

**Keywords:** dielectric, ferroelectric, graphene, barium titanate, polyvinylidene fluoride, capacitors

## Abstract

To address the limitations of piezoelectric polymers which have a low dielectric constant andto improve their dielectric and ferroelectric efficiency for energy storage applications, we designed and characterized a new hybrid composite that contains polyvinylidene fluoride as a dielectric polymer matrix combined with graphene platelets as a conductive and barium titanite as ceramic ferroelectric fillers. Different graphene/barium titanate/polyvinylidene fluoride nanocomposite films were synthesized by changing the concentration of graphene and barium titanate to explore the impact of each component and their potential synergetic effect on dielectric and ferroelectric properties of the composite. Results showed that with an increase in the barium titanate fraction, dielectric efficiency ofthe nanocomposite was improved. Among all synthesized nanocomposite films, graphene/barium titanate/polyvinylidene fluoride nanocomposite in the weight ratio of 0.15:0.5:1 exhibited thehighest dielectric constant of 199 at 40 Hz, i.e., 15 fold greater than that of neat polyvinylidene fluoride film at the same frequency, and possessed a low loss tangent of 0.6. However, AC conductivity and ferroelectric properties of graphene/barium titanate/polyvinylidene fluoride nanocomposite films were enhanced with an increase in the graphene weight fraction. Graphene/barium titanate/polyvinylidene fluoride nanocomposite films with a weight ratio of 0.2:0.1:1 possessed a high AC conductivity of 1.2 × 10^−4^ S/m at 40 Hz. While remanent polarization, coercive field, and loop area of the same sample were 0.9 μC/cm^2^, 9.78 kV/cm, and 24.5 μC/cm^2^·V, respectively. Our results showed that a combination of graphene and ferroelectric ceramic additives are an excellent approach to significantly advance the performance of dielectric and ferroelectric properties of piezoelectric polymers for broad applications including energy storage.

## 1. Introduction

Dielectric and piezoelectric polymer materials have attracted significant attention to replace piezoelectric ceramic materials used in various applications such as medical, automotive industry, and consumer electronics dueto their lightweight, flexibility, low-cost and low-acoustic impedance, and high piezoelectric constant [1,2,3]. Apart from biomedical applications, they have recently been extensively explored for energy applications including energy storage devices like transistors, nano-generators, capacitors, actuators, and electromechanical transducers, etc. [4,5,6,7]. The dielectric parameters of dielectric and piezoelectric polymer materials are also very important in new, very accurate capacitance and inductance measurement methods (sensors) as shown in previous reports [8,9]. An ideal dielectric material for these applications must possess high dielectric constant (ε′), lowenergy dissipation (tanδ), low dielectric loss (ε′′), and high mechanical strength which are possible to achieve with polymer materials which are mechanically stable, flexible, and easy to process and make devices. The limitation of piezoelectric polymers is a low ɛ′, usually less than 10, which is not ideal for capacitor applications. To overcome this limitation, a number ofceramic polymer composites were explored as dielectric fillers in different energy storage devices. Common ceramic fillers being reinforced in polymers are calcium copper titanate/polyimide (CaCu₃Ti₄O₁₂/PI), lead zirconatetitanate (Pb[Zr_x_Ti_1−x_]O₃), barium titanate (BaTiO_3_), and titania (TiO_2_) [10,11,12,13]. Rao et al. synthesized a ceramic polymer composite containing lead magnesium niobite-lead titanate/barium titanate (PMN-PT/BaTiO_3_) embedded in modified epoxy. Dielectric constant of the resulting composite was 110 while its tanδ was 0.016 at 10 kHz [14]. Ravindra et al. synthesized barium titanate/polyvinylidene fluoride (BaTiO_3_/PVDF) composite possessing ε′ of 7 and tanδ as low as 0.03 [15]. Sugumaran et al. synthesized TiO_2_ composites with polyvinyl alcohol (PVA) and poly(methyl methacrylate) (PMMA) with ε′ of 24.6 and 26.8 for both composites, respectively [13]. Although ceramic materials bear high ε′, low ε′′ and excellent ferroelectric characteristics, a high loading of ceramics in polymer matrix results in limitations like high weight, low flexibility, poor mechanical strength due to poor filler matrix bonding, and flocculation of ceramic granules [16].

Another strategy to address the limitations of piezoelectric polymers is to add a small amount of conducting fillers into the polymer matrix to achieve high ɛ′. Some conducting fillers including multiwalled carbon nanotubes (MWCNT) [17], nano iron oxide (Fe_3_O_4_) [18], silver (Ag) nanoparticles [19], and graphene, [20] etc. have been explored. Although larger particles of conducting fillers are very close to each other in polymermatrix, their conducting paths are not well aligned due to the presence of the polymer matrix. Conducting nanoparticles are considered to be more efficient in enhancing dielectric properties of the composite than micro-sized fillers of the same material [21]. The conducting filler network in the matrix embellish ε′. ε′′ of the composite also increases, thus, suppressing its applications [22,23].

To further advance the properties of dielectric polymer materials, the combination of both ceramic and conducting fillers has been explored to design ternary polymer composites. Yao et al. increased ε′ of BaTiO_3_/PVDF composites by adding MWCNT filler. Synthesized three-phase composite showed enhanced dielectric efficiency with ε′ of 151 and tanδ of 0.08 at 100 Hz [17]. Another ternary composite PVDF/Ag/BaTiO_3_ reported by Zhang et al. possessed ε′ equal to 40.8 and tanδ of 0.055 at 100 Hz showing improvement in dielectric efficiency because it used Ag with a higher conductivity [19]. Wang et al. reported the synthesis of a three-phase composite of surface functionalized graphene with BaTiO_3_ and PVDF possessing ε′ and tanδ equal to 65 and 0.35, respectively at 1 MHz that demonstrated a promising potential of combined graphene/ceramic additives to advance performance of polymer dielectrics [24]. Although studies have been carried out with three-phase composites comprising polymer and both conductive and ceramic fillers, most of the previous studies were focused on materials and fundamental aspects and there was lack of exploration and advancement in their performance for energy storage applications. This field has scope for further investigations to improve dielectric performance of dielectrics. Moreover, ferroelectric properties of three-phase flexible dielectric composites, which play a key role in efficiency of dielectrics in various devices, have not been well explored. Although the effect of graphene and ceramic fillers on dielectric performance of polymers composites has been previously investigated, their combined effect on enhancing ferroelectric properties of dielectrics is still not explored and needs further investigations.

In this paper, we present the use of graphene as a conducting element to design three-phase graphene/barium titanate/polyvinylidene fluoride (G/BT/PVDF) nanocomposites with high ε′, low tanδ, and enhanced ferroelectric properties and explore performance for energy storage and capacitors applications. To demonstrate this concept, we selected graphene as the conducting filler because of its controllable conductive properties, size and 2D unique structure, high mechanical strength, and high electrical conductivity (σ) [25,26]. Among all ceramics, barium titanate (BT) was chosen because of its environmentally friendly nature being lead-free, low manufacturing cost, fairly high ε′, and because it is a ferroelectric ceramic [27]. Polyvinylidene fluoride (PVDF) was used as a base matrix because of its highest ε′ among polymers, efficient energy storage properties, and stable piezoelectric characteristics [28,29]. This work aims to explore the influence of both additives, graphene and BT, on dielectric and ferroelectric performance, and to find their optimized ratio and possible synergy. One challenging problem to solve is to optimize the dispersion of conductive graphene sheets with BT and polymer matrix. For that purpose, we explored many different options for dispersion, graphene and BT dosages, and explored their influence on dielectric and ferroelectric properties of the composite. The dielectric efficiency of synthesized G/BT/PVDF nanocomposite films was characterized by evaluating their ε′, tanδ, AC conductivity, and electric modulus. Synthesized G/BT/PVDF nanocomposite films not only showed good dielectric properties but also enhanced ferroelectric characteristics which are depicted by polarization-electric field (PE) curves showing the significant potential of this approach for designing advanced piezoelectric polymer with ferroelectric properties that can be used for broad applications including energy storage. Our synthesized G/BT/PVDF nanocomposite films have the advantages of flexibility and piezoelectric properties of PVDF, conductivity, mechanical strength of graphene, high ε′, and enhanced thermal stability of BT. It may be a good addition to dielectrics in energy storage devices.

## 2. Materials and Methods

### 2.1. Materials

Commercial natural graphite rocks (Uley, Eyre Peninsula, South Australia, Australia) were purchased from an Australian mining site. Potassium permanganate (97% KMnO_4_, Sigma-Aldrich, Gillman, Australia), hydrochloric acid (35% HCl, Chem-Supply, Gillman, Australia), sulfuric acid (98% H_2_SO_4_, Chem-Supply, Gillman, Australia), phosphoric acid (85% H_3_PO_4_, Chem-Supply, Gillman, Australia), hydrogen peroxide (30% H_2_O_2_, Chem-Supply, Gillman, Australia), N,N–Dimethylforamide (99.8% DMF, Chem-supply, Gillman, Australia), barium titanate(IV) (< 100 nm particle size, 99% BaTiO_3_, Sigma-Aldrich, St. Louis, MO, USA) and polyvinylidenefluroride (PVDF, Sigma-Aldrich, St. Louis, MO, USA) were of analytical grade and were used without further purification.

### 2.2. Synthesis of Graphene/Barium Titanate/Polyvinylidene Fluoride (G/BT/PVDF) Nanocomposite Films

Graphene oxide (GO) was synthesized by improved method [30], and it was further reduced to graphene [31]. Ternary G/BT/PVDF nanocomposite was synthesized by a solution mixing technique in w/w fraction. First, a measured quantity of graphene and BT was dissolved in 10 mL of N,N–Dimethylforamide (DMF) and ultrasonicated by using aprobesonicatorBranson Digital Sonifier 450, Branson Ultrasonics Corporation, Danbury, CT, USA, until the graphene was completely dispersed. In the meantime, 1 g of PVDF was dissolved in 20 mL of DMF at 80 °C. After the PVDF was completely dissolved in DMF, a solution of graphene/barium titanate (G/BT) was dissolved by stirring at 80 °C for 45 min. To make a free-standing film, the mixture was cast on a petri dish, oven dried at 80 °C until the solvent was completely evaporated leaving behind a film of G/BT/PVDF nanocomposite.

**Figure 1:**
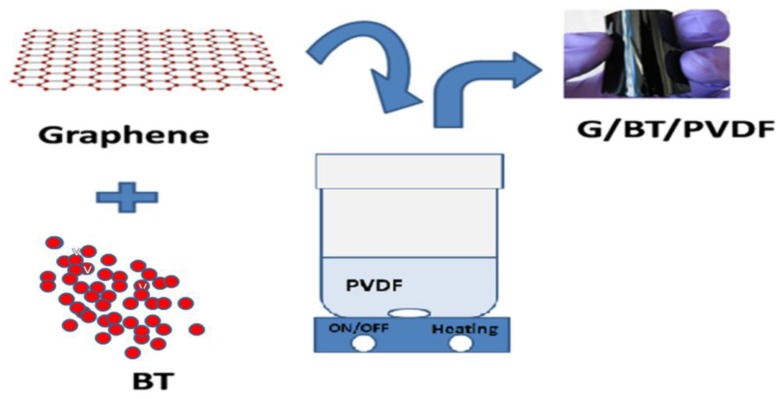
Schematic illustration of the synthesis of graphene/barium titanate/polyvinylidene fluoride (G/BT/PVDF) nanocomposite films.

Different G/BT/PVDF nanocomposite films of uniform thickness of 550 μmwere synthesized by the solution casting technique using different graphene content while keeping the concentration of BT (100 mg) and PVDF (1 g) constant. We explored several different G/BT ratios to analyzetheir influence on dielectric and ferroelectric properties and found an optimized composite formulation for future work. After characterization and calculating ε′ and tanδ of the above synthesized films, asuitable fraction of graphene with high ε′ and low tanδ (G:BT:PVDF = 0.15:0.1:1) was selected and further samples were synthesized with varying concentrations of BT while keeping the concentration ratios of graphene and PVDF (0.15:1) constant. The scheme of the designed composites with their key results is presented in Figure 1. Table 1 shows the weight fractions of all three components in different G/BT/PVDF nanocomposite films with their sample codes.

### 2.3. Characterizations

All samples including PVDF and ternary G/BT/PVDF nanocomposite films were analyzed to study their morphology by field emission scanning electron microscopy (FESEM, Quanta 450, FEI, Hillsboro, OR, USA). Structural investigations were made by using X-ray diffraction (XRD, 600 Miniflex, Rigaku, Akishima, Japan) and Raman spectroscopy by using Raman microscope (LabRamHR Evolution, Horiba Ivon Yvon Technology, Kyoto, Japan) equipped with LabSpec 6 software (Horiba Ivon Yvon Technology, Kyoto, Japan) A 532 nm laser line from He–Ne source was used for excitationand 600 grooves per mm grating was used. Acquisition time was 5 s and the accumulation time was 3 s. Fourier transform infrared (FTIR) spectroscopy (Nicolet 6700, Thermo Fisher, Waltham, MA, USA) in transmittance mode and range 400–4000 cm^−1^ was used to identify the functional groups of synthesized nanocomposite films. Thermal stability was studied by using a thermal gravimetric analyzer (TGA, Q500, TA Instruments, New Castle, DE, USA) under air where the samples were heated from room temperature (RT) to 900 °C at a heating rate of 10 °C min^−1^. Dielectric characterization of the synthesized G/BT/PVDF nanocomposite films was carried out using precision impedance analyzer (Wayer Kerr, 6500B, West Sussex, UK) at RT between 20 Hz to 1 MHz. Films were plated between copper electrodes and measurements were done at RT. Ferroelectric characterization of all samples was made by using Radiant Technologies Multiferroic testing system. Samples were placed between copper electrodes at RT.

## 3. Results and Discussion

### 3.1. Characterization of Graphene/Barium Titanate/Polyvinylidene Fluoride (G/BT/PVDF) Nanocomposite Films

**Figure 2:**
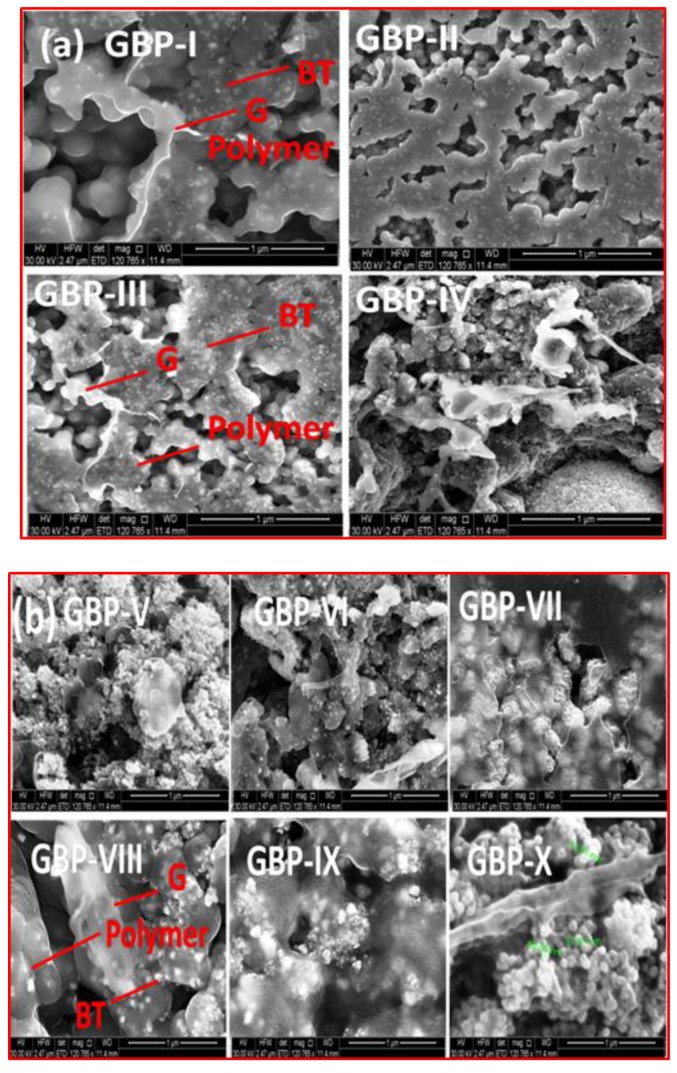
Field emission scanning electron microscopy (FESEM) images of (**a**) G/BT/PVDF nanocomposite films with different graphene fraction and (**b**) G/BT/PVDF nanocomposite films with different barium titanate (BT) fraction.

Morphology of the synthesized G/BT/PVDF nanocomposite films was characterized by FESEM as shown in Figure 2a,b. FESEM images show that graphene sheets are not well connected; rather these are segregated by BT nanoparticles and PVDF matrix. Both graphene sheets and BT particles are well dispersed in the polymer matrix. The size of BT nanoparticles was measured by FESEM, and it was found to be 50–70 nm. BT nanoparticles are decorated on graphene sheets as well as in the polymer matrix. As shown in Figure 2a, graphene sheets become more prominent as the weight fraction of graphene increases from GBP-1 (50 mg) to GBP-IV (200 mg). Figure 2b shows that with increasing BT content, BT nanoparticles start to aggregate. Aggregations are more prominent in GBP-X with maximum BT fraction (700 mg).

**Figure 3:**
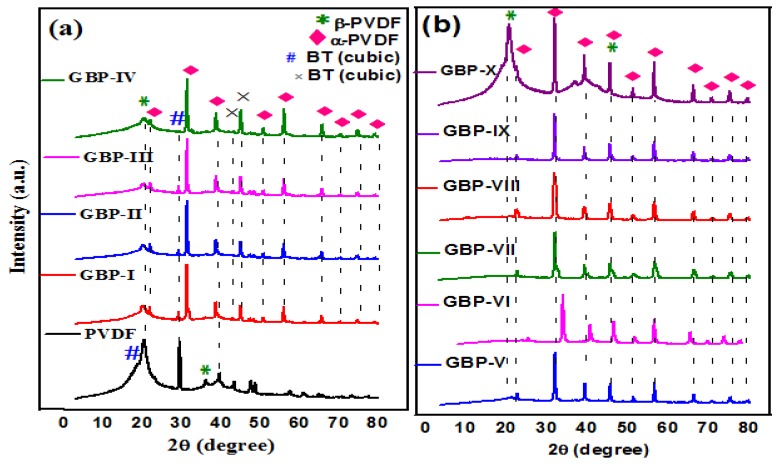
X-ray diffraction (XRD) pattern of (**a**) neat polyvinylidene fluoride (PVDF) and G/BT/PVDF nanocomposite films with different graphene fraction (**b**) G/BT/PVDF nanocomposite films with different BT fraction.

XRD analysis was carried out to investigate the crystal structure of the PVDF film and synthesized G/BT/PVDF nanocomposite films. Figure 3a,b show the XRD patterns of each film. The XRD peak at 20.1° corresponding to (100), (200) shows the existence of beta (β) phase of the PVDF, while the peak at 18.3° (021) and 29.1° (−211) corresponds to the alpha (α) phase of PVDF. However, peaks belonging to α phase of PVDF are missing in G/BT/PVDF nanocomposites depicting that PVDF has been converted to the β phase with the addition of graphene and BT fillers [32,33]. Strong diffraction peaks at 22.3°, 31.5°, 38.9°, 45.2°, 50.9°, 56.2°, 65.8°, 70.4°, 74.9°, and 79.1° corresponding to (100), (110), (111), (200), (210), (211), (220), (212), (301) and (311), respectively, according to JCPDS no. 831880 are assigned to BT nanoparticles. A peak at 2θ = 45.2° for (200) shows the cubic structure of the BT nanoparticles [34]. However, in GBP-I, GBP-II, GBP-III, and GBP-IV, this (200) peak splits into two peaks (200) and (002) depicting that higher graphene fraction causes disorder and formation of a tetragonal structure of BT [35]. This splitting is not shown in XRD spectra of samples with a high concentration of BT as there is a lower fraction of graphene to causedistortion of the BT structure. The peak at 2θ = 45.2° for (200) is intensified with an increasing BT fraction from GBP-V to GBP-X. Unfortunately, the peak for graphene was not observed in the XRD spectra of all nanocomposites, possibly due to the segregation of graphene sheets in the presence of BT and PVDF as is evident from FESEM images in Figure 2a,b. Therefore, to confirm the presence of graphene, the Raman spectroscopy of selected samples (GBP-III and GBP-IX) was carried out.

**Figure 4:**
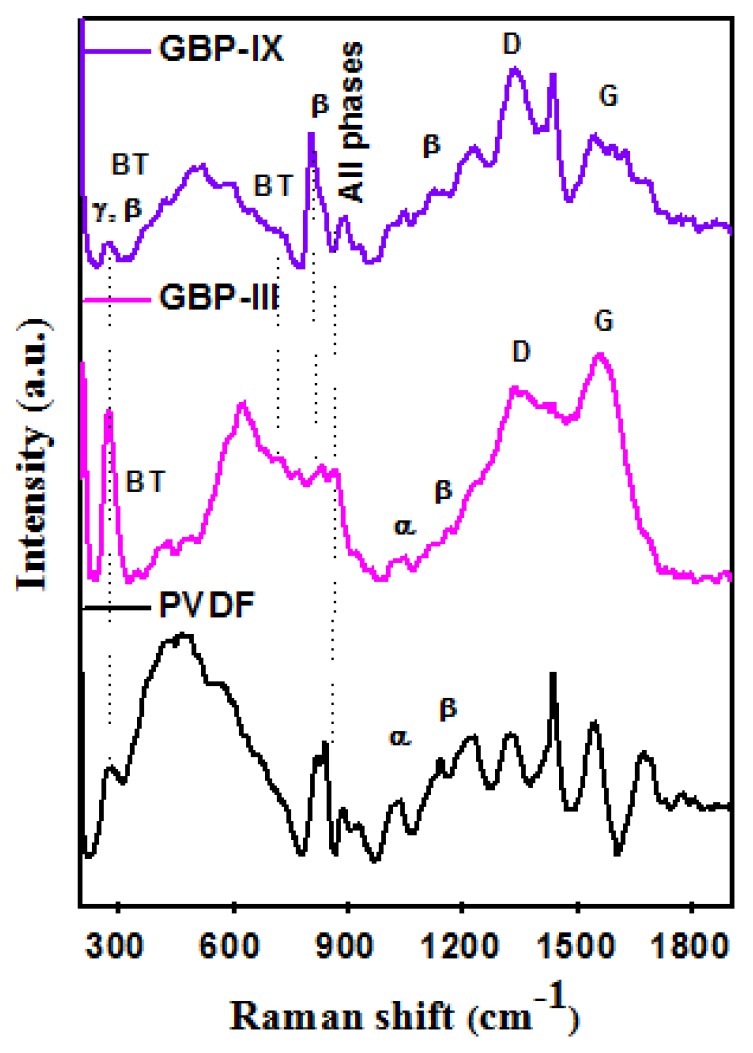
Raman spectra of PVDF, GBP-III, and GBP-IX nanocomposite films.

Raman spectra of neat PVDF film, GBP-III, and GBP-IX were recorded to confirm the presence of graphene, BT, and PVDF in synthesized G/BT/PVDF nanocomposite films. Results of Raman spectroscopy are in accordance with those of XRD except that it also shows graphene bands. Raman spectra are shown in Figure 4. Bands at 718 cm^−1^ belong to the tetragonal structure of BT nanoparticles [36]. A very sharp band at 275 cm^−1^ corresponds to beta (β) and gamma (γ) phases of PVDF. The GBP-III nanocomposite shows a band at 839 cm^−1^ confirming that PVDF is in β phase. The well-defined D and G bands of graphene appeared at 1333 cm^−1^ and 1557 cm^−1^ in the Raman spectra of GBP-III while these bands appear at 1333 cm^−1^ and 1546 cm^−1^, respectively in Raman spectra of GBP-IX. The intensity ratio of D and G bandsId/Ig was calculated to be 0.82 and 1.02 for GBP-III and GBP-IX, respectively. Id/Ig ratio shows that defects in graphene structure are more pronounced in GBP-III than in GBP-IX as the former has relatively higher graphene fraction than later [32].

**Figure 5:**
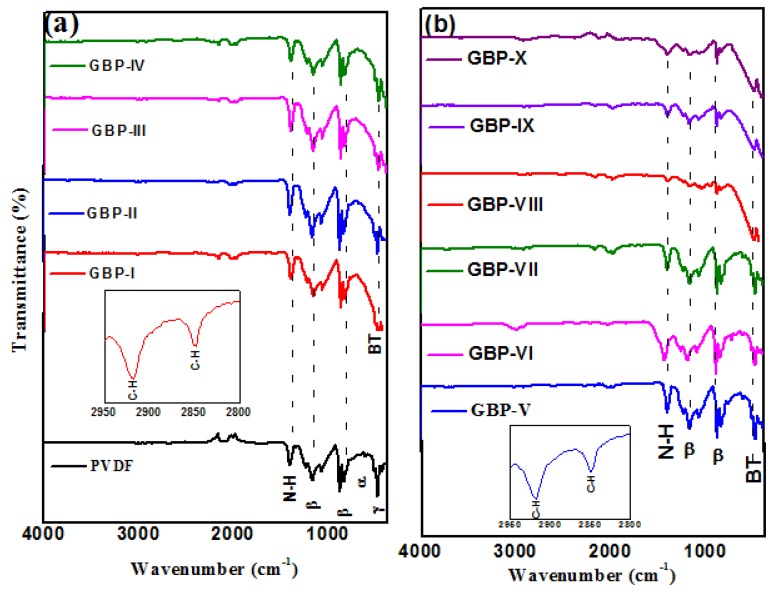
Fourier transform infrared (FTIR) spectra of (**a**) neat PVDF and G/BT/PVDF nanocomposite films with differentgraphene fractions and (**b**) G/BT/PVDF nanocomposite films with different BT fraction.

FTIR spectra of all synthesized samples were recorded to further confirm the formation of G/BT/PVDF nanocomposites. FTIR spectra of G/BT/PVDF nanocomposites are shown in Figure 5. Characteristic peak of BT nanoparticles was observed at 470 cm^−1^ [37]. The peak at 825 cm^−1^ suggests the presence of β phase of PVDF [38]. The peak at 1160 cm^−1^ is also indicating β phase PVDF. Two weak peaks at 2849 cm^−1^ and 2917 cm^−1^ correspond to C–H stretching of PVDF [32]. A characteristic stretching peak for carbonyl group (C = O) and alcoholic group (C–O) at 1743 cm^−1^ is present [39]. But it is very weak showing that graphene is embedded in PVDF and graphene sheets are not continuous as shown by FESEM images in Figure 2a,b. The N–H stretching peak at 1400 cm^−1^ is indicative of entrapped remnants of DMF in nanocomposite films [32].

**Figure 6:**
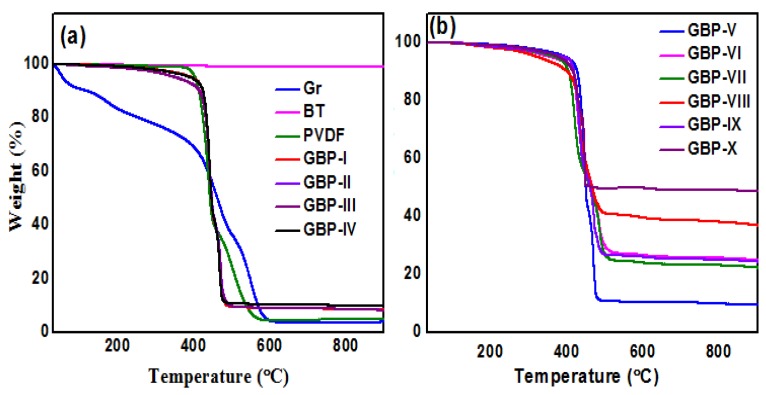
Thermal gravimetric (TG) curves of (**a**) neat PVDF and G/BT/PVDF nanocomposite films with different graphene fraction; (**b**) G/BT/PVDF nanocomposite films with different BT fractions.

Thermal gravimetric analyses (TGA) of graphene, BT, neat PVDF film, and all synthesized G/BT/PVDF nanocomposite films were carried out to study their thermal stability. TG curves show that BT is stable over a high temperature range; even at 900 °C it is stable and does not decompose. Graphene decomposes with a rise in temperature and decomposes up to 95% around 600 °C. It was observed that incorporation of BT in the polymer improves thermal stability of the polymer from GBP-V to GBP-X. It is due to the high surface area contact of BT with the polymer matrix that results in the restriction of PVDF degradation [40]. As BT fraction increases, thermal stability of G/BT/PVDF nanocomposite also increases as is evident by TG curves in Figure 6a,b.

### 3.2. Dielectric Studies of G/BT/PVDF Nanocomposite Films

**Figure 7:**
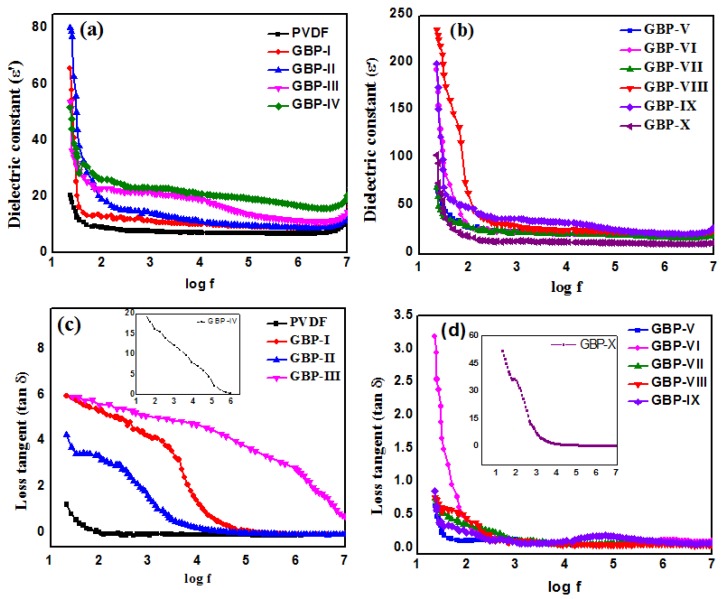
Dielectric constant (ε′) of (**a**) neat PVDF and G/BT/PVDF nanocomposite films with different graphene fraction (**b**) G/BT/PVDF nanocomposite films with different BT fractions.Loss tangent (Tanδ) of (**c**) neat PVDF and G/BT/PVDF nanocomposite films with different graphene fraction (**d**) G/BT/PVDF nanocomposite films with different BT fraction.

Dielectric constant (ε′) of neat PVDF and all synthesized G/BT/PVDF nanocomposites was plotted against frequency as shown in Figure 7a,b. Dielectric response of materials is frequency dependent. Higher ε′ at lower frequency range corresponds to Maxwell-Wagner-Sillar (MWS) polarization simply describing interfacial polarization at insulator–conductor interface. It explains that enhancement of ε′ of the heterogeneous composite is due to entrapment of free mobile charges at the insulator–conductor interface [41]. A decrease in ε′ at a higher frequency range is associated with the polarization relaxation phenomenon. When the frequency is high, dipoles may not undergo rapid changes with changing electric field. It causes losses in the equilibrium state which, in turn, causes a reduction in ε′ [15,42]. A sharp decrease inε′ value is observed at a lower frequency that becomes steady at a higher frequency. Stable ε′ at a higher frequency is attributed to the micro capacitor structural model [23]. Figure 7a shows that ε′ of G/BT/PVDF nanocomposite films increases with a high content of graphene while keeping BT fraction constant. After the incorporation of graphene in the PVDF matrix, an increase in ε′ is due to the formation of many microcapacitors. Graphene provides for microcapacitors’ conducting pathways by acting as conducting electrodes and PVDF provides insulating pathways [23]. From GBP-I to GBP-IV, ε′ of nanocomposites enhance with increasing graphene contents. From GBP-III to GBP-IV, ε′ rises sharply near the percolation threshold as predicted by percolation theory. The results are in agreement with numerous literature findings concerning composites involving conducting fillers in a polymer matrix [23,41]. Although ε′ of GBP-IV is much greater than that of GBP-III, it can be further improved by increasing the graphene fraction. However, with an increase in ε′, tanδ also enhances many folds as is evident from Figure 7c. For this reason, we selected GBP-III for further experiments. As can be seen, both ε′ and tanδ of G/BT/PVDF increase with an increase in BT content. This rise is due to structure modifications of the nanocomposites. When BT fraction is low, ε′ is low. However, with an increase in BT fraction, BT nanoparticles start to flocculate and these BT aggregates penetrate into PVDF matrix. As the concentration of BT increases, size of flocculates also grows, as is evident in FESEM images in Figure 2b. It results in the formation of a more polarizable network of BT and better dispersion of BT in PVDF causing an increase in ε′ of the nanocomposite films. This higher ε′, after high loading of BT in nanocomposites, suggests that synthesized G/BT/PVDF nanocomposite is a suitable candidate to be used in capacitors [43]. Results show that both graphene and BT fillers improve ε′ of the PVDF. This observation is quite reasonable as both fillers have the ability to enhance ε′ of PVDF matrix. However, both fillers follow different mechanisms to elevate ε′. Graphene increases ε′ of the polymer by following percolation theory [44]. While an increase in ε′ after adding BT can be explained by the effective medium theory [45]. ɛ′ improved dramatically and reached up to 199 for GBP-VIII; that is about 15 times larger than that of pure PVDF (ε′ = 13) at 40 Hz as shown in Figure 7b. At the same frequency, tanδ of GBP-VIII is 0.6, as shown in Figure 7d. With a rise in frequency,ε′ decreases but at about 1 kHz it becomes steady and constant. ε′ becomes constant even at the very high frequency of 1MHz, showing that G/BT/PVDF nanocomposite films are stable over a long range of frequency.

At lower frequency, tanδ is low which may be due to the presence of ionizable hydroxyl group (OH) at the heterogeneous interface as well as the fact that an extendedtime is available for diploes to collide [46]. Not only ε′ but also tanδ of G/BT/PVDF nanocomposites rise with the increase of both graphene and BT fractions. Tanδ of neat PVDF and all synthesized G/BT/PVDF nanocomposite films is shown in Figure 7c,d. Increasing the conducting graphene filler fraction in the PVDF matrix increases the leakage current causing a rise in tanδ [46]. On the other hand, an increase in tanδ by increasing BT fraction is due to large aggregates of BT as shown in Figure 2b. This results in an electric field where dipoles may collide, thus, producing heat [47]. Synthesized nanocomposites (GBP-VIII and GBP-IX) have a high ε′, and tanδ is less than 2.5, which aredesirable characteristics for capacitor applications [48]. So, our material is well suited to be used as a dielectric medium in high energy storage capacitors. A comparison of ɛ′ and tanδ of G/BT/PVDF nanocomposite films and previously reported dielectric is summarized in Table 2. Values of ε′ and tanδ of dielectrics are given at high frequencies. If we compare these values with that of our synthesized G/BT/PVDF nanocomposite fims, we see that G/BT/PVDF nanocomposite films have still high ε′ of 22.5 even at a high frequency of 1MHz which is still considered better than values of other reported materials as values of other materials are reported at lower frequency than 1 MHz and it is explained that with rise of frequency value of ε′ decreases but G/BT/PVDF nanocomposite films has maintained ε′ of 22.5 still reasonable to be used as dielectric in capacitors as shown in Table 2. At the same time, tanδ of G/BT/PVDF nanocomposite films is still very low as shown in Table 2.

**Figure 8:**
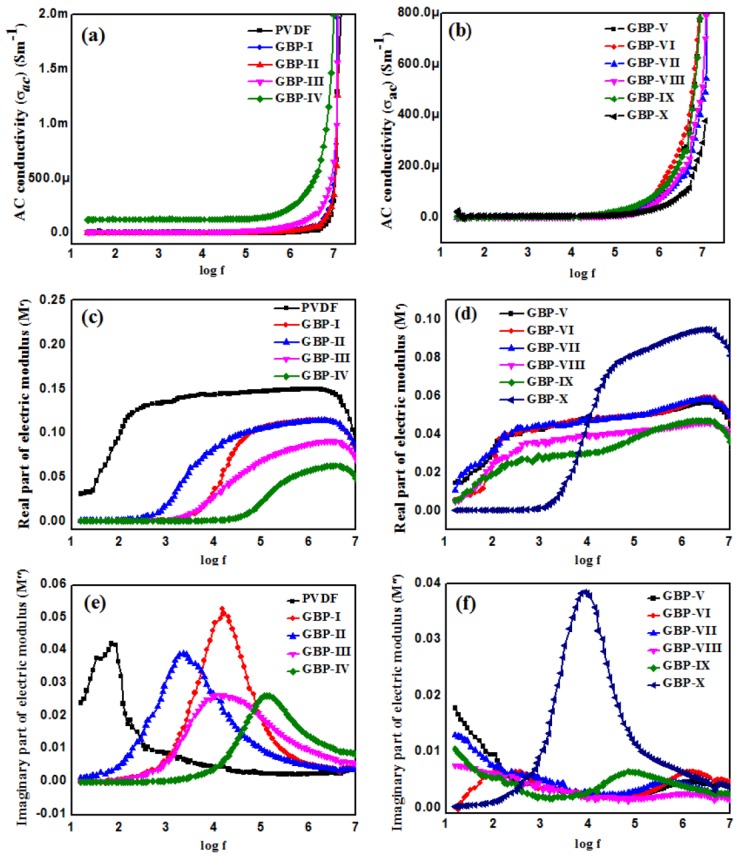
AC conductivity (σ*_ac_*) of (**a**) neat PVDF and G/BT/PVDF nanocomposite films with different graphene fraction (**b**) G/BT/PVDF nanocomposite films with different BT fraction. Real part of the electric modulus (M′) of (**c**) neat PVDF and G/BT/PVDF nanocomposite films with different graphene fraction (**d**) G/BT/PVDF nanocomposite films with different BT fraction. Imaginary part of the electric modulus of (**e**) neat PVDF and G/BT/PVDF nanocomposite films with different graphene fraction (**f**) G/BT/PVDF nanocomposite films with different BT fraction.

AC conductivity (σ*_ac_*) of PVDF and all nanocomposite films is shown in Figure 8a,b. Results show that the GBP-IV nanocomposites filled with maximum high conducting graphene contents possess the highest σ*_ac_* of 1.2 × 10^−4^ (S/m) at 40 Hz. While a further increase in graphene might enhance the σ*_ac_* and ε′, a further rise in graphene content was not carried out due to the expected increase in tanδ. However, when BT content was further increased, it caused segregation of graphene sheets as shown in FESEM images in Figure 2b. The conductivity of the nanocomposites increases from GBP-V to GBP-IX, although it is less than σ*_ac_* of GBP-IV. GBP-IX possesses the highest conductivity among nanocomposites with varying BT contents (GBP-V to GBP-X). It is due to the distance between graphene sheets reducing with increasing BT content to a certain extent. Here, tunneling conductance occurs through neighboring graphene sheets [52].

The dielectric modulus formalism is used to study the electrical transport within the dielectrics. Figure 8c,d illustrate the plots of real (M′) and imaginary part (M′′) of the complex modulus (M*) as a function of frequency for all samples. With a change in frequency, electrical transport in the material changes, the electric modulus also changes. M′ increases with increasing frequency and reaches a maximum limit termed the asymptotic value (M∞) indicating that nanocomposites are very capacitive in nature [53]. Figure 8e,f shows M′′ plotted against frequency. It rises and reaches a maximum value M′′max then decreases with an increase in frequency [53].The reason is that charge carriers are mobile over a long distance until M′′max, and then charge carriers are only mobile for short distances and are confined to potential wells [54].

### 3.3. Ferroelectric Studies of G/BT/PVDF Nanocomposite Films

**Figure 9:**
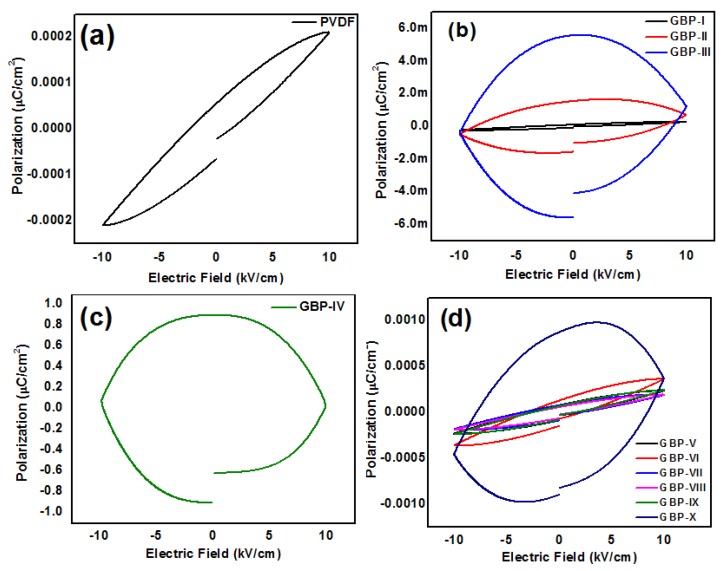
Polarization electric field (P-E) hysteresis loop of (**a**) neat PVDF (**b**) GBP-I, GBP-II, GBP-III (**c**) GBP-IV (**d**) G/BT/PVDF nanocomposite films with different BT fractions.

The polarization electric field (P-E) hysteresis loop was studied to further analyze thegeneration of charge in the synthesized nanocomposite films. Analyses were carried out at RT. The thickness of each film was 550 μm, and the area of the electrode was 0.786 cm^2^. Results of ferroelectric studies of all samples are shown in Figure 9. Remanent polarization (Pr) of neat PVDF film was 5.5 × 10^−5^ μC/cm^2^. While coercive field (Ec) and loop area of PVDF film were 1.29 kV/cm and 0.00146 μC/cm^2^·V, respectively, as shown in Figure 9a.

It is evident from the ferroelectric analysis that by increasing graphene content while keeping BT fraction constant, ferroelectric properties are enhanced. Pr, Ec, and loop area of GBP-IV with maximum graphene fraction (200 mg) are 0.9 μC/cm^2^, 9.78 kV/cm, and 24.5 μC/cm^2^·V, respectively. It is the highest among samples with different graphene content as shown in Figure 9b,c. As the graphene fraction increases, energy dissipation is also increased as depicted in the previous section. Energy dissipation is responsible for the separation of charge and voltage signals resulting in loops with a considerable area of the curves [53]. Area of the curve defines the charge storage ability of the material; the greater the area of the curve, the greater the charge storage ability of the material [55]. An increase in Pr by the addition of conducting graphene filler is attributed to hetero-polarization caused by conducting filler-polymer interaction. In addition, it is due to the formation of β phase of PVDF [56]. The formation of β phase of PVDF is evident by XRD, Raman spectra, and FTIR spectra as explained earlier. By keeping the graphene fraction constant and varying the concentration of BT, the ferroelectric properties of nanocomposites are also improved. For GBP-X, Pr, Ec, and loop area were found to be 0.00087 μC/cm^2^, 8.4 kV/cm, and 0.025 μC/cm^2^·V, respectively. Although these values are not much larger when compared to GBP-IV, they are still much better than that of neat PVDF. An increase in ferroelectric properties by increasing the BT content can be explained as follows:When BT content is low, there are fewer mobile charge carriers. Thus, energy dissipation is also low, and loop area is also low, but when BT fraction is increased, the number of charge-carrying particles is also increased. It results in increased energy dissipation and an enhanced loop area [12]. A slight deviation appeared in P-E hysteresis loop of GBT-VI. It may be due to the agglomeration of BT particles in the polymer matrix. The results show that conductive graphene filler is more effective in increasing ferroelectric properties of piezoelectric polymer than ferroelectric ceramic (BT) filler.

## 4. Conclusions

In this paper, we designed and explored a concept to enhance dielectric and ferroelectric properties of piezoelectric polymers for energy storage applications by combining conductive graphene and ferroelectric ceramic (BT) additives. New hybrid composite films of G/BT/PVDF were synthesized with variations in dosage and ratio of graphene and BT to explore their influence on dielectric (ε′ and tanδ) and ferroelectric (Pr, Ec, and loop area) values. In the first set of experiments, we kept the concentration of BT and PVDF constant and changed the concentration of graphene, until it reached a value with high ε′ and low tanδ. The optimized graphene fraction which showed good dielectric properties was selected for synthesis of another series of G/BT/PVDF nanocomposite films. In the second experiment, the concentration of BT was increased, andthe concentration of graphene and PVDF was fixed. GBP-VIII nanocomposite film with graphene, BT, and PVDF fraction (0.15:0.5:1) was found to have the highest ε′ values (199 at 40 Hz) and lowesttanδ (0.6) among all prepared samples, that is, it is highly suitable as a dielectric candidate for capacitors. Regarding the ferroelectric properties of prepared G/BT/PVDF nanocomposite films, results showed that GBP-IV (G:BT:PVDF = 0.2:0.1:1) has the best ferroelectric properties (Pr, Ec, and loop area of GBP-IV are 0.9 μC/cm^2^, 9.78 kV/cm, and 24.5 μC/cm^2^·V, respectively) among neat PVDF and all synthesized G/BT/PVDF nanocomposites. Our results demonstrate that the combination of graphene and ferroelectric ceramic additives is an excellent approach to enhance significantly the performance of dielectric and ferroelectric properties of piezoelectric polymers and can be used for broad applications, specifically, to be suitable for high performing energy storage and supercapacitors.

## Figures and Tables

**Table 1:** Sample Codes and composition of prepared graphene/barium titanate/polyvinylidene fluoride (G/BT/PVDF) nanocomposite films.

Sample Code	Weight of Graphene(mg)	Weight of BT (mg)	Weight of PVDF (mg)	Weight Ratio of G:BT:PVDF
GBP-I	50	100	1000	0.05:0.1:1
GBP-II	100	100	1000	0.1:0.1:1
GBP-III	150	100	1000	0.15:0.1:1
GBP-IV	200	100	1000	0.2:0.1:1
GBP-V	150	200	1000	0.15:0.2:1
GBP-VI	150	300	1000	0.15:0.3:1
GBP-VII	150	400	1000	0.15:0.4:1
GBP-VIII	150	500	1000	0.15:0.5:1
GBP-IX	150	600	1000	0.15:0.6:1
GBP-X	150	700	1000	0.15:0.7:1

**Table 2:** Comparison of dielectric constant (ε’) and loss tangent (tanδ) of G/BT/PVDF nanocomposite film with previously reported dielectric materials.

Dielectric Material	Frequency (Hz)	Dielectric Constant (ɛ’)	Loss Tangent (tanδ)	References
PBCNCs-3D	1000/100	16.2	0.15	[49]
PMMA/rPANI@rGO	1000	40	0.12	[50]
PVA/TiO_2_	1000	24.6	0.1–1	[12]
PMMA/TiO_2_	1000	26.8	0.1–0.8	[12]
PMN-PT/BaTiO_3_/Epoxy	10000	110	0.016	[13]
PVDF/Graphite	1000	4.5 × 10^7^	229	[23]
BTNTs/PVDF	100	47.05	0.1	[51]
G/BT/PVDF	40	199	0.6	This work
G/BT/PVDF	10^6^	22.5	0.05	This work
